# Supplementary material for: Foliar Diseases and the Associated Fungi in Rice Cultivated in Kenya
Source: Plants (Basel). 2022 May 7;11(9):1264. doi: 10.3390/plants11091264 (PMC9105481; doi:10.3390/plants11091264)
Supplement: Supplementary file 1 [file plants-11-01264-s001.zip › plants-1645567-Supplementary.pdf]

Supplementary Table S1. Correlation between foliar disease components and agronomic traits of rice based on a survey conducted in Busia and Kisumu Counties of Kenya in 2019

	Blast severity	Blast incidence	Brown spot incidence	Blast infection rate	Age of the crop	Plant height (cm)
**Blast incidence**	0.9566 (<0.0001)					
**Brown spot incidence**	0.3437(0.0109)	0.342 (0.0122)				
**Blast infection rate**[1]	0.1302(0.7027)	0.4989(0.1183)	-0.7189(0.1430)			
**Brown spot infection rate**[2]	0.1471(0.9764)	-0.0506(0.4077)	-0.0461(0.893)	-0.3488(0.2931)		
**Age of the crop**	0.1506(0.3349)	0.246(0.1163)	0.2203(0.1506)	.		
**Plant height (cm)**	0.203(0.1619)	0.2628(0.0711)	0.3494(0.012)	.	0.609(<0.0001)	
**lodging (0-3)**	0.1576(0.2794)	0.2194(0.1341)	0.2611(0.0671)	.	0.4394(0.0032)	0.4929(<0.0001)

[1]Brown spot and [2]blast infection rates are based on proportion of samples with detectable *Cochliobolus miyabeanus* or *M. oryzae* using PCR. Within each cell: Spearman's rho correlation coefficient outside the bracket and the *p*-value within the bracket.

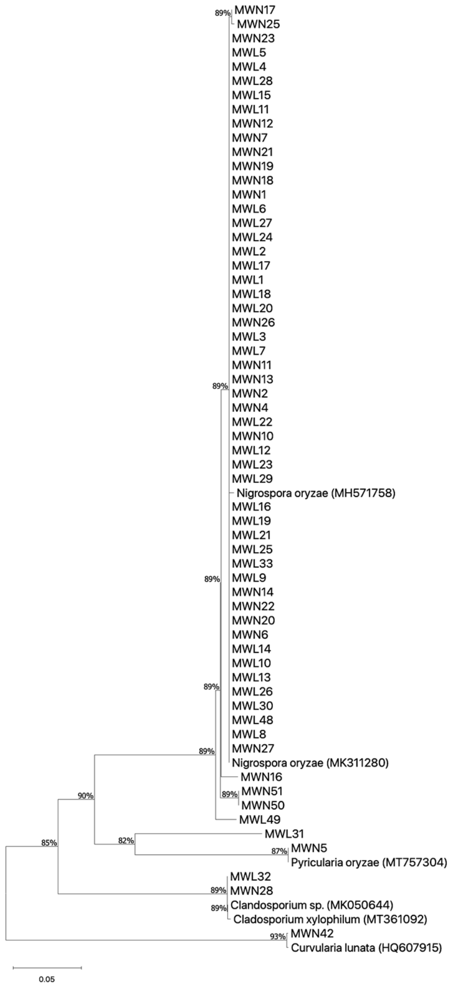

Supplementary Figure S1. Genetic relationship for isolates of *Nigrospora oryzae* from Kirinyaga, Kenya. Isolates of *Pycularia oryzae (Magnaporthe oryzae)* (MWN5 and MT757304), *Cladosporium sp* (MWL32, MWN28, MK050644 and MT361092), and *Cuvularia lunata* (MWN2 and HQ607915) were included as outgroups. The Neighbor-joining tree was generated using nucleotides of the internally transcribed spacer (ITS) of the ribosomal RNA.
